# Pilot study of a novel multi‐functional noninvasive prenatal test on fetus aneuploidy, copy number variation, and single‐gene disorder screening

**DOI:** 10.1002/mgg3.597

**Published:** 2019-02-14

**Authors:** Yuqin Luo, Bei Jia, Kai Yan, Siping Liu, Xiaojie Song, Mingfa Chen, Fan Jin, Yang Du, Juan Wang, Yan Hong, Sha Cao, Dawei Li, Minyue Dong

**Affiliations:** ^1^ The Department of Obstetrics and Gynecology, Women's Hospital, School of Medicine Zhejiang University Hangzhou Zhejiang China; ^2^ The Center for Prenatal and Hereditary Disease Diagnosis, Department of Obstetrics and Gynecology, Nanfang Hospital Southern Medical University Guangzhou China; ^3^ Department of Gynecology and Obstetrics Wuhan Medical and Health Center for Women and Children Wuhan Hubei China; ^4^ Department of Prenatal Diagnosis Nanping Maternity and Child Health Hospital Nanping Fujian China; ^5^ Annoroad Gene Technology Co., Ltd Beijing China

**Keywords:** chromosome aneuploidy, CNVs, NIPT, single‐gene disorder, target sequencing

## Abstract

**Background:**

The noninvasive prenatal testing (NIPT) has been successfully used in the clinical screening of fetal trisomy 13, 18, and 21 in the last few years and researches on detecting sub‐chromosomal copy number variations (CNVs) and monogenic diseases are also in progress. To date, multiple tests are needed in order to complete a full set of fetus disorder screening, which is costly and time consuming. Therefore, an integrated 3‐in‐1 NIPT approach will be in great demand by routine clinical practice in the near future.

**Methods:**

We designed a target capture sequencing panel with an associate bioinformatics pipeline to create a novel multi‐functional NIPT method and we evaluated its performance by testing 22 clinical samples containing aneuploidy, CNV, and single‐gene disorder. Chromosomal aneuploidy and CNV were detected based on the Z‐value approach, whereas single‐gene disorder was identified by using the “pseudo‐tetraploid” model to estimate the best‐suited genotype for each locus.

**Results:**

The performance of this newly constructed 3‐in‐1 system was promising. We achieved a 100% detection rate for chromosomal aneuploidies (7/7), a 100% diagnosis rate for fetus CNVs larger than 20 Mb (3/3), and an 86.4% accuracy for single‐gene disorder screening (19/22).

**Conclusion:**

For the first time, we showed that it is possible to use just a single NIPT test to detect three distinct types of fetus disorder and laid a foundation for developing a cheaper, faster, and multi‐functional NIPT method in the future.

## 1 INTRODUCTION

Each year, nearly 8 million children are born with a severe birth defect worldwide, in which 3.2 million are estimated to have lifelong disabilities (Christianson, Howson, & Modell, 2006). In China, the incidence of birth defects is 5.6%, which is equivalent to 90,000 new cases every year (PRC Ministry of Health, 2013). Although the exact etiologies of birth defects still remain unclear, the importance of genetic disorder such as chromosome aneuploidy, sub‐chromosomal copy number variations (CNVs), and single‐gene mutation have been recognized (Webber et al., 2015).

Conventional clinical methods for detecting the aforementioned genetic defects are mostly invasive, for example chorionic villus sampling and amniocentesis, which may promote the risk of fetal miscarriage and intrauterine infection (Tabor et al., 1986). However, after the discovery of cell‐free fetal deoxyribonucleic acid (cffDNA) in maternal peripheral blood (Lo et al., 1997) and the development of next‐generation sequencing (NGS), a new era of noninvasive prenatal test (NIPT) have emerged. This technology has now been widely used in the clinical screening of trisomy disorders such as Patau, Edwards, and Down syndrome (Chiu et al., 2008; Fan, Blumenfeld, Chitkara, Hudgins, & Quake, 2008). In addition, researches on adapting NIPT to detect other types of fetal gene disorder are also underway. In 2011, Peters et al. (2011) successfully identified a 4.2 M deletion on fetal chromosome 12 from cffDNA and proved that it is feasible to detect fetal sub‐chromosomal CNVs with the noninvasive method. Subsequently, between 2015 and 2017, a number of reports have demonstrated that fetal single‐gene disorders such as congenital adrenal hyperplasia, Duchenne muscular dystrophy, and type I Gaucher disease could also be identified via the noninvasive NGS approach (New et al., 2014; Xu et al., 2015; Zeevi et al., 2015).

Despite the technical capabilities of detecting chromosome aneuploidy, CNVs, and single‐gene disorder from cfDNA in maternal blood, pregnant women would currently still need to take multiple noninvasive tests in order to complete the full set of fetal genetic disorder screening, and each test requires 5–10 ml of peripheral blood, on average 20 days of turn‐around time (TAT), and a sum of examination fee. It is therefore costly and time consuming for the wide use in routine clinical practice and integrating three tests into one single examination would be very beneficial. However, one of the major difficulties for the integration is how to incorporate the fetus single‐gene mutation analysis into aneuploidy and CNVs detection workflow since the latter two mainly rely on the unique reads count and Z‐value, whereas the former one is mostly a haplotype‐based method (Allen, Young, & Bowns, 2017; Hui et al., 2017). Moreover, for aneuploidy and CNVs screening, the coverage can be as low as just 0.01× depending on the CNVs resolution, but at least several hundred sequencing depth was required for the fetal SNP identification. So far, there have not been any published researches managed to successfully overcome the two obstacles.

In this pilot study, we attempted to tackle this problem by developing a target sequencing approach with corresponding bioinformatics workflow. We designed a target capture panel that covers 8,731 regions across all chromosomes and mutation hotspots in six genes of the three common Chinese single‐gene disorder, β‐thalassemia, hearing‐impairment, and phenylketonuria. For the single‐gene disorder detection, we chose to use the “pseudo‐tetraploid” approach (Yin et al., 2018) instead of haplotype method to estimate the best‐suited genotype for each locus. We also carried out a single‐blind test using this newly constructed 3‐in‐1 noninvasive fetus genetic disorder screening system on 22 clinical samples containing aneuploidy, CNVs and single‐gene disorder to evaluate its feasibility and the results were promising. Therefore, despite the imperfection and in need of further improvement, here, for the first time, we showed that it is possible to use just one NGS test to detect three distinct types of fetus gene disorder and laid a foundation for developing a cheaper, faster, and optimized multi‐functional noninvasive prenatal test in the future.

## 2 MATERIALS AND METHODS

### 2.1 Ethical compliance

The research was approved by the ethics committee from Nanfang Hospital, Southern Medical University, Guangzhou, China (No. NFEC‐2014‐048) and the Women's Hospital, School of Medicine, Zhejiang University, Zhejiang, China (No. 20160104). All procedures performed in studies involving human participants were in accordance with the ethical standards of the institutional and/or national research committee and with the 1964 Helsinki Declaration and its later amendments or comparable ethical standards.

### 2.2 Panel design

**Figure 1:**
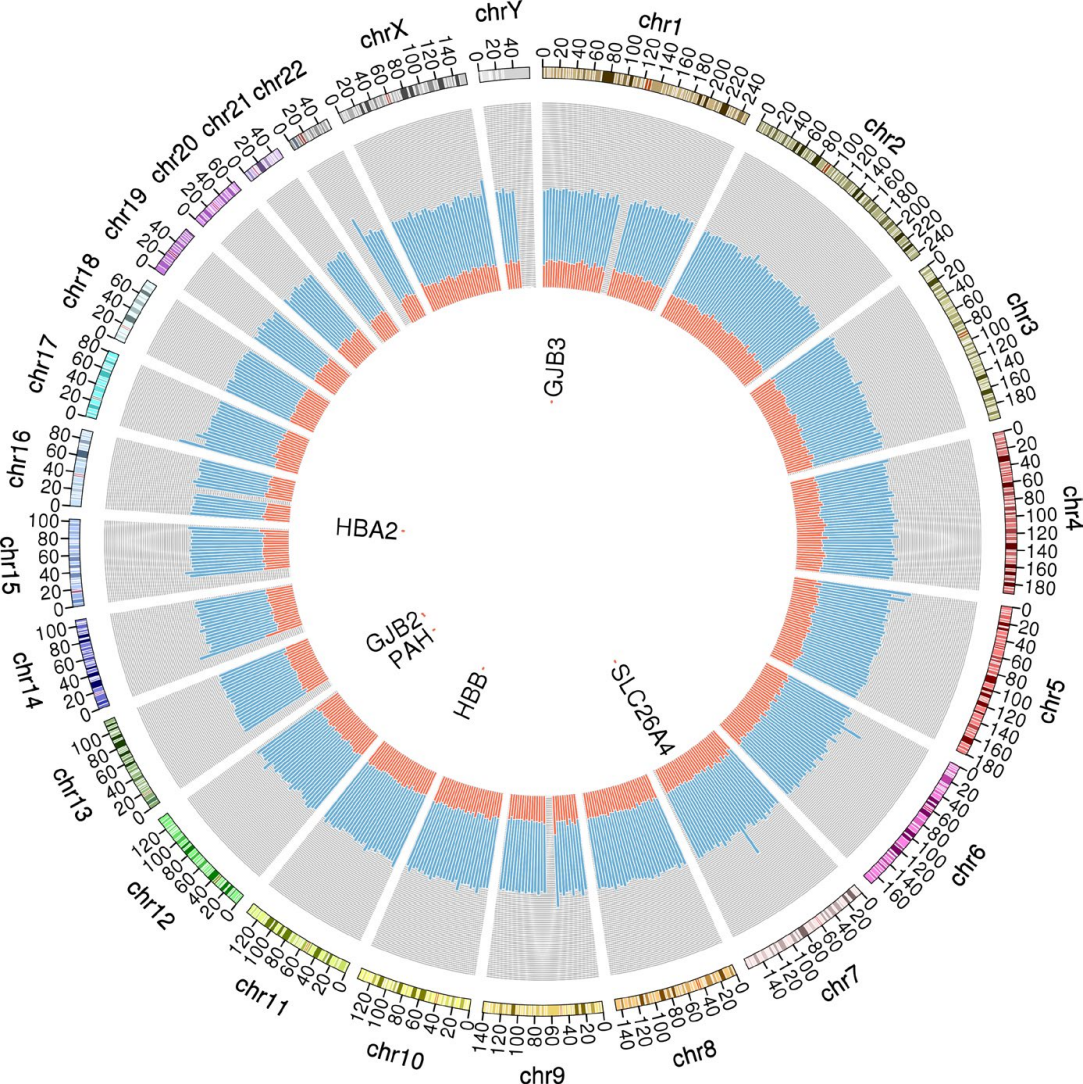
Circos diagram of the target region capture panel. Chromosome ideograms (genome build hg19) are shown in the outmost ring and oriented pter to qter in a clockwise direction. Other tracks, located on the gray background, from inside to outside are the genes tested for the single‐gene disorder, palindrome content (red; range, 0–1), and GC content (light blue; range, 0–1)

The conventional NIPT method developed in 2008 is a shallow depth whole genome sequencing (WGS) approach without target selection, and this is considered to be problematic and a waste of reads, since substantial amount of sequencing reads were from highly repetitive regions and regions with high GC contents on the genome. Targeted capture sequencing, on the other hand, could provide a great degree of freedom for picking specific regions with fewer repeats, consistent GC contents and pathogenic SNP loci (Figure 1).

In this study, a custom target region capture panel was designed to cover 8,731 regions. Of which, 8,171 regions with equal‐length are uniformly scattered on 22 autosomes and sex chromosomes and the remaining 560 regions are unequal length and consist of common mutation hotspots for single‐gene disorder β‐thalassemia, hearing‐impairment, and phenylketonuria (Figure 1).

### 2.3 Sample information

A total of 90 singleton pregnancies were enrolled from Women's Hospital School of Medicine Zhejiang University (Hangzhou, China) in this study after receiving the fully informed consent from the individuals. In which, peripheral blood of 68 healthy pregnant women were used to construct a standard negative background, and the rest of 22 clinical samples with fetal genetic disorder were single‐blindly tested. All maternal blood samples were drawn before amniocentesis or chorionic villus sampling using EDTA‐k_2_ vacuum tube and cfDNA was extracted and stored at −80°C within 4 hr after collection. The genetic status of the 22 samples were determined through karyotyping, amniotic fluid NGS (Qi et al., 2018), or cord blood sanger sequencing and their paired cfDNA samples, stored at −80°C, were re‐numbered and sent out for testing. The 22 samples contained seven cases of chromosomal aneuploidies, three cases of fetus CNVs, five cases of hearing‐impairment, two cases of phenylketonuria, and five cases of β‐thalassemia.

### 2.4 DNA preparation and sequencing

For every sample, 5 ml of maternal peripheral blood was centrifuged at 1600 *g* for 10 min to separate the plasma, then it was transferred to a new tube and centrifuged at 4°C for 10 min at 16,000 *g* to remove the remaining cells. One millilitre of plasma was used to extract cell‐free DNA using the MagMAX™ Cell‐Free DNA Isolation Kit (Applied Biosystems USA) and the rest of plasma was stored in −80℃ for further use. Approximately 10 ng of cell‐free DNA could be obtained from each sample to construct sequencing library. The steps include end repair, 3ʹ adenylation, adaptor ligation, and 11 cycles of PCR amplification using common primers and tagging primers. After each step, the Agencourt AMPure XP‐nucleic acid purification kit (Beckman Coulter, USA) was used for nucleic acid retrieval.

Qubit 3.0 (Applied Biosystems) was used to evaluate the library concentration before mixing them together with equal quantity. Subsequently, 1 µg of the library mixture was hybridized with the SeqCap EZ Probes oligo pool (Roche Nimblegen, USA) from the target capture panel at 47°C for 72 hr, followed by a second round of PCR amplification in accordance with the standard procedure of the SeqCap EZ Probes handbook. The captured DNA fragments were purified using the Agencourt AMPure XP‐nucleic acid purification kit and evaluated using Agilent 2100 Bioanalyzer (Agilent, USA) and quantitative PCR. Finally, the libraries were sequenced using Nextseq550AR (Annoroad Gene Technology, China) by following standard instructions.

### 2.5 Data analysis

The customized data analysis workflow is an integrated bioinformatics pipeline that consists of three functions, autosomal and sex chromosome aneuploidies identification, sub‐chromosomal CNVs detection, and single‐gene disorders screening.

#### 2.5.1 Autosomal and sex chromosome aneuploidies

In this study, 68 euploid samples were used to construct background library. In brief, unique reads that aligned to the human reference genome (GRCh37.p13) were extracted, followed by counting the number of reads in 7,631 autosomal regions (overlap >1 bp). The counted regional depths were then standardized to 1 million reads and mean and standard deviation of each chromosomal region were calculated. For testing samples, the above procedures were repeated for each chromosome. The mean of all chromosomal regions was used to calculate the Z‐value and each possible chromosomal aneuploidy was detected by comparing the Z‐value with the normal range obtained from the background database. For sex chromosomes, the median and mean of 88 regions of the Y chromosome were used to differentiate the gender of unborn fetuses. Twenty‐five samples with less than 5% N and more than 50% base sequence quality >30 were selected for building the background baseline. The analysis procedure was the same as that of autosomes, except that the number of regions used during read number normalization was 8,171.

#### 2.5.2 Chromosomal CNVs detection

To determine the fetus CNV status, the same 68 euploid samples were used to construct a background CNV database. In short, the detection process includes counting the reads number of each region, normalization, calculating the Z‐value of each region and merging chromosomal windows using *HaarSeg* algorithm (Ben‐Yaacov & Eldar, 2008). The candidate CNV regions were selected based on the average Z‐value of merged windows using [−0.7, 0.7] as the normal value range, followed by comparing the median value of the candidate CNVs regions with the 95% confidence interval of the background regions’ median value to call for the true CNVs.

#### 2.5.3 Single‐gene disorders screening

In this study, the genotype of each locus in the sample was considered as a “pseudo‐tetraploid” since it is a mixture of maternal and fetal DNA in plasma. To determine the fetal genotype from cfDNA sequencing results, a previously described novel approach called Pseudo Tetraploid Genotyping (PTG) was used (Yin et al., 2018). Briefly, the probability of a pseudo tetraploid genotype can be calculated by multiplying the probability of the unique genotype to itself or any two genotypes. Out of nine possible mother‐fetus combinations, only seven (“AAAA”, “AAAB”, “ABAA”, “ABAB”, “ABBB”, “BBAB”, and “BBBB”) could be recovered from the mother‐fetus genotype mixture in maternal plasma, where “A” stands for the reference base, and “B” is the most frequent base other than “A”. The first two letters of each pseudo tetraploid represent the maternal genotype and the last two letters represent the fetal genotype. Among the seven combinations, only four mother‐fetus genotype combinations (“AAAB”, “ABAA”, “ABBB”, and “BBAB”) can theoretically be used to calculate fetal fraction.

The correct inference of the “pseudo‐tetraploid” genotype relies heavily on the accurate estimation of fetal concentration. To optimize the fetal concentration and obtain the best genotype of each locus, the EM algorithm and Bayesian model were used respectively and the relationship between the genotype and fetal concentration was used to re‐estimate the fetal concentration. The analysis was then repeated until reaching the point where the difference between the last two estimated fetal concentrations was below 0.001 and this concentration was used to calculate the best‐suited genotype of each locus. Finally, the disease status was determined based on the estimated locus genotype.

## 3 RESULTS

### 3.1 Panel design and process optimization

**Figure 2:**
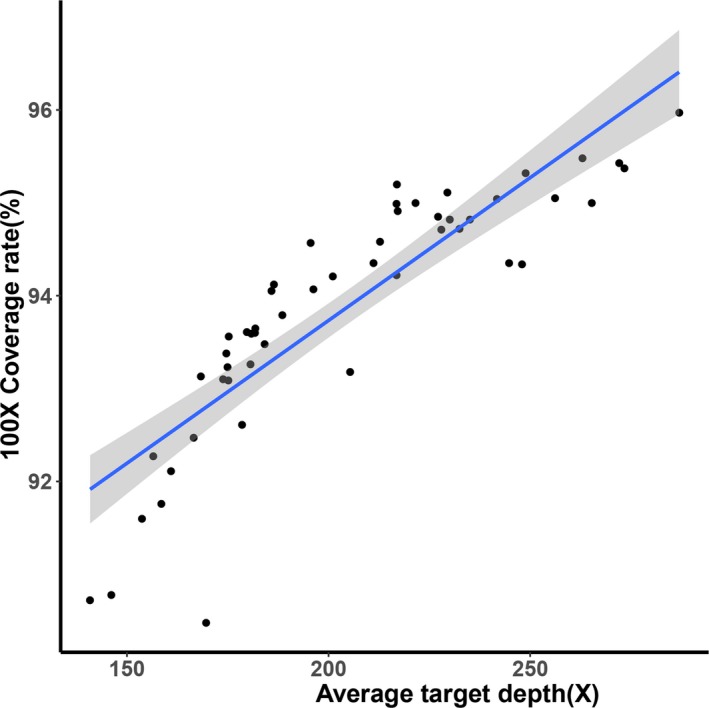
Scatter plot of average target depth against 100× coverage rate in the background database. Unique reads of 22 chromosomes in 68 samples were used to build a standard comprehensive negative background database. The scatter plot showed that the average target depth was in positive correlation with the 100× coverage rate (%). Solid line represents the trend line of linear regression

**Figure 3:**
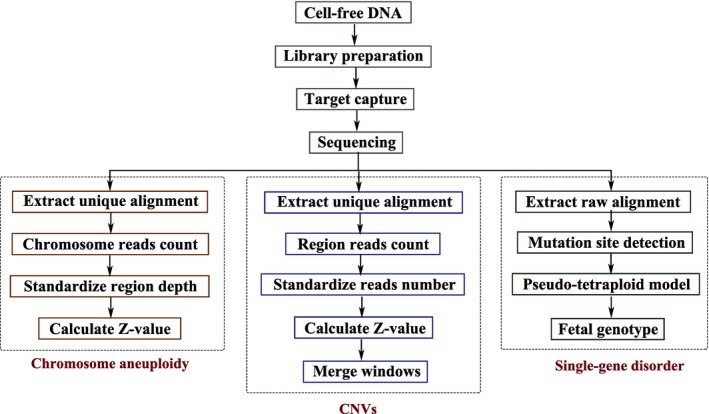
Flowchart of the analysis process. The complete analysis process includes cfDNA extraction, library preparation, target capture, sequencing, and bioinformatics analysis. For chromosomal abnormality and CNV detection process, the number of reads were standardized through comparison with extracted high‐quality unique reads, and Z‐score was then calculated to obtain the results; for the single‐gene disorder detection process, single nucleotide variant (SNV) detection was performed using GATK to compare the unique results. The pseudo‐tetraploid model was used to estimate the best fetal DNA concentration and the best genotype was obtained. Finally, the results were annotated and the corresponding disease loci were identified

The panel we designed in this study contains a consistent level of GC content in order to significantly decrease the impact of GC bias in target hybridization and sequencing. Therefore, variations caused by high GC content would no longer be an interference factor. In addition, by selecting regions with equal‐length and similar thermodynamic property, we could use the potentially uniformed distribution of reads coverage to build a standard comprehensive negative background library (Figure 2). The three‐in‐one bioinformatics workflow of chromosomal abnormality, CNVs and single‐gene disorder analysis was shown in Figure 3.

### 3.2 Performance evaluation

#### 3.2.1 Autosomal and sex chromosome aneuploidies

**Figure 4:**
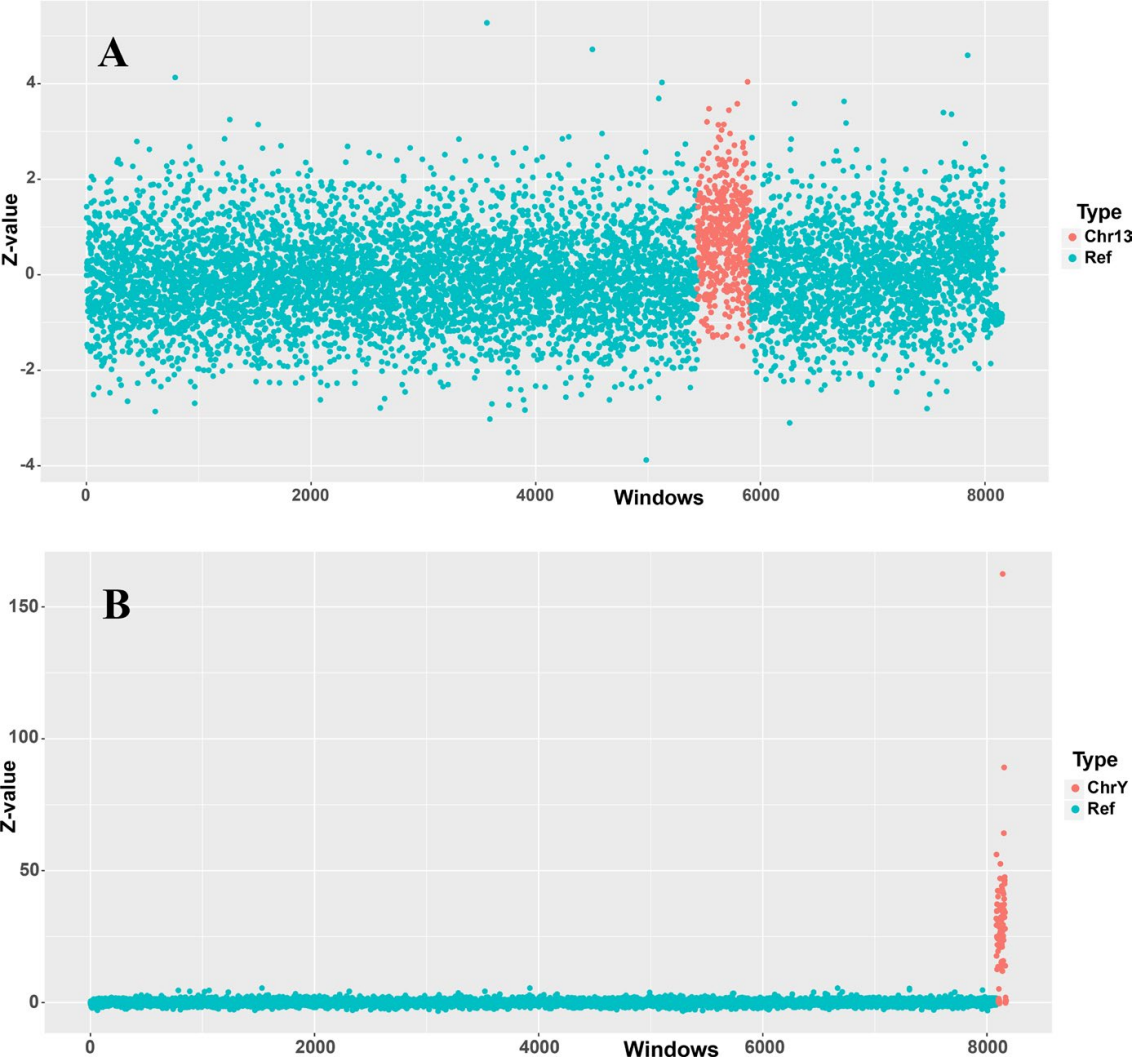
Distribution scatter plots of trisomy 13 and 47, XXY. Representations of autosome trisomy and sex chromosomal abnormality. Z‐value was used to indicate the presence of chromosomal abnormality in two samples. (a) Sample AWD01Y1401479‐1‐51 had a much higher Z‐value in chromosome 13 region, this indicates that the infant carries trisomy 13; (b) Chromosome Y of sample AWD01Y1401491‐1‐63 showed a much higher Z‐value and this represent a 47, XXY karyotype

Pregnant women aged between 25 and 41 (mean: 33.3, *SD *±7.39) were enrolled in this research and their peripheral blood were collected between 12 to 23 gestational weeks (mean: 18.1, *SD *±3.4). Fetal fraction, which is crucial for avoiding false negative results, was ranged between 16% and 22% with an average of 18.72%. Chromosome aneuploidy was detected in 7 of 22 samples (Figure 4) and the test reached a 100% sensitivity and specificity. The Z‐values of seven positive samples are listed in Table 1.

**Table 1:** Comparison of chromosome aneuploidy identified using karyotype and targeted capture sequencing with seven pregnant women

Sample ID	GA	MW (kg)	Gestation (week + day)	Serum screening	Karyotype	NIPT targeted capture sequencing								
						Fetal gender	FF (%)	ATD(X)	Chromosome Z‐values^a^					Result
									Chr13	Chr18	Chr21	ChrX	ChrY	
AWD01Y1401479‐1‐51	35	66	21 + 0	NA	T13	Female	19.76	195.32	5.53	0.11	−0.44	0.2	−0.42	T13
AWD01Y1401480‐1‐52	26	55	12 + 2	NA	T18	Female	16.90	149.09	−0.66	3.81	−0.4	−2.68	45.28	T18
AWD01Y1401481‐1‐53	41	50	17 + 1	High risk	T18	Female	18.14	185.02	−0.36	3.68	−0.04	0.39	−0.42	T18
AWD01Y1401482‐1‐54	40	51	23 + 1	ND	T21	Male	18.83	154.08	−0.42	−0.09	4.18	0.27	−0.42	T21
AWD01Y1401483‐1‐55	26	54	18 + 1	High risk	T21	Female	21.24	213.41	−0.48	−0.53	5.41	−5.45	81.56	T21
AWD01Y1401490‐1‐62	25	59	18 + 1	Low risk	45, X	45, X	19.86	239.56	0.11	−0.83	−0.83	−3.6	−0.42	X0
AWD01Y1401491‐1‐63	40	56	17 + 0	High risk	47, XXY	47, XXY	16.32	269.11	0.22	1.56	0.01	0.51	64.15	XXY

Notes

GA, gestational age; MW, maternal weight; NA, not available; ND, not detected; FF, fetal fraction; ATD(X), average target depth(X); Chr, chromosome.

a Only listed the values of targeted chromosomes.

#### 3.2.2 Chromosomal CNVs detection

**Figure 5:**
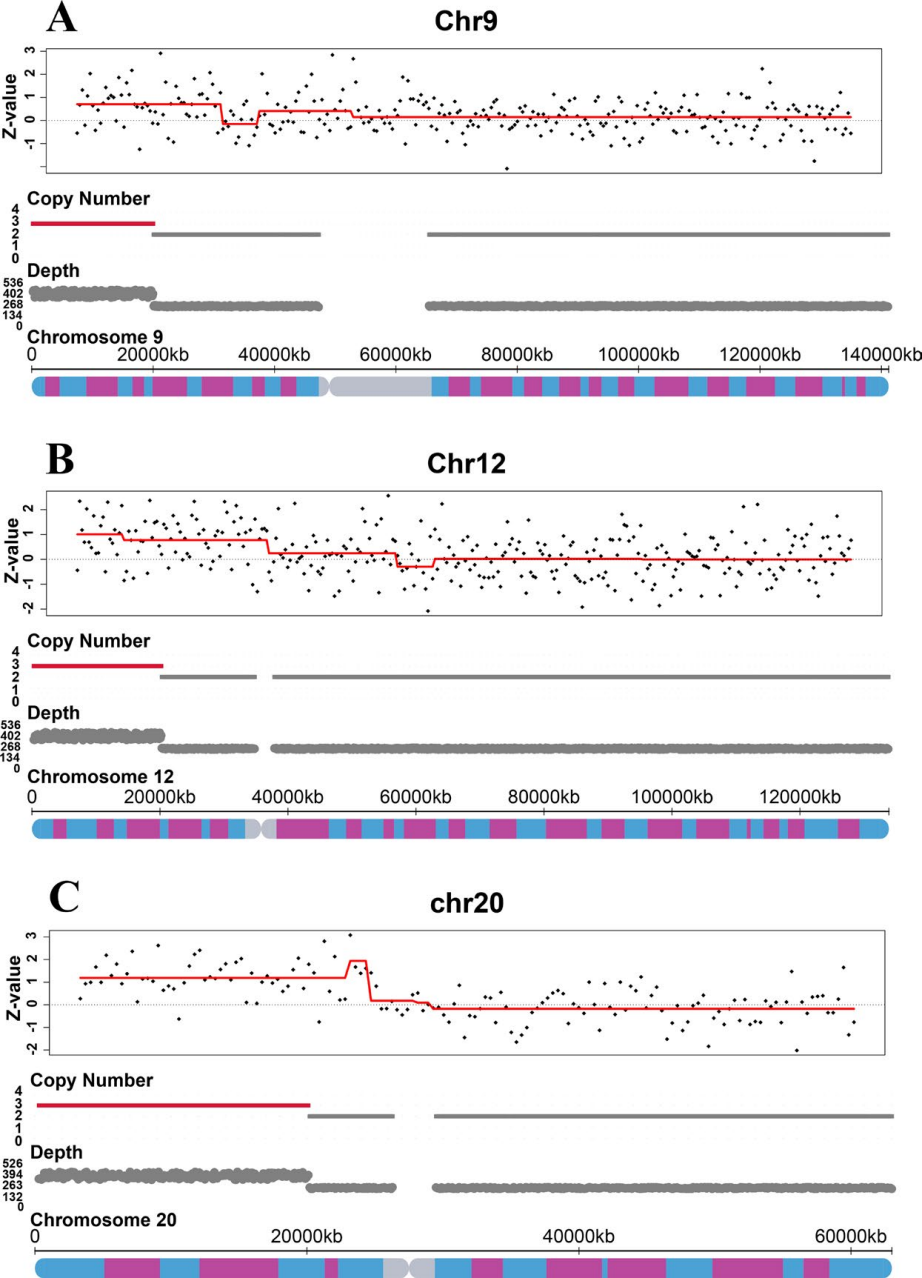
Z‐value and copy number indicate the presence of CNVs in three samples. (a) Chromosome 9 of sample AWD01Y1401487‐1‐59 showed a duplication in short arm between bands 9p24.3 and 9p21.3; (b) Chromosome 12 of sample AWD01Y1401486‐1‐58 had a duplication in short arm between bands 12p13.33 and 12p13.31, and bands 12p13.31 and 12p11.21; (c) Chromosome 20 of sample AWD01Y1401488‐1‐60 displayed a duplication in short arm between bands 20p13 and 20p11.23

The *HaarSeg* algorithm was used to calculate the Z‐value of each region, and CNVs were called only after meeting the following three criteria. 1. At least 20 continuous regions have the resolution larger than 10 Mb; 2. The mean Z‐value is outside the range −0.7 to 0.7; 3. The median Z‐value is outside the range of −2 to 2. Our results showed that in 22 samples, all three fetuses with CNVs over 20 Mb in size were successfully identified, but when compared with the amniotic fluid NGS results, the lengths of CNV that we detected on chromosome 9 in sample AWD01Y1401487‐1‐59 and on chromosome 12 in AWD01Y1401486‐1‐58 were shorter due to the choice of target capture region selection (Figure 5, Table 2). In addition, three CNVs with the size less than 2 M were too short to be detected by this method.

**Table 2:** Comparison of copy number variants (CNVs) identified using next‐generation sequencing of amniotic fluid and targeted capture sequencing of maternal peripheral blood

Sample ID	GA	MW (kg)	Gestation (week + day)	Serum screening	CNVs size	Amniotic fluid result	NIPT targeted capture sequencing					
							Fetal gender	FF (%)	ATD(X)	Median Z‐value of region	CNVs size	Chromosome location
AWD01Y1401487‐1‐59	29	63	15 + 3	High risk	38.55 M	seq [GRCh37]dup(9)(p24.3p21.3) chr9:g. 210001_38760000dup	Male	16.99	270.53	3.37	21 M	seq [GRCh37]dup(9)(p24.3p21.3) chr9:g.1_21000000dup
					2 M	seq [GRCh37]dup(16)(q24.2q24.3) chr16:g. 88260001_90260000dup				ND	ND	ND
					0.65 M	seq [GRCh37]dup(19)(q11q12) chr19:g. 28360001_29010000dup				ND	ND	ND
AWD01Y1401486‐1‐58	39	62	24 + 6	NA	1.05 M	seq [GRCh37]dup(10)(q25.1) chr10:g. 109560005_110610004dup	Female	17.96	278.99	ND	ND	ND
					37.8 M	seq [GRCh37]dup(12)(p13.33 q11) chr12:g. 60001_37860001dup				2.60	8 M	Seq [GRCh37]dup(12)(p13.33p13.31) chr12:g.1_8000000dup
										2.77	23.5 M	seq [GRCh37]dup(12)(p13.31q11.21) chr12:g. 8000001_31500000dup
AWD01Y1401488‐1‐60	27	58	18 + 3	Low risk	20 M	seq [GRCh37]dup(20)(p13p11.22) chr20:g. 60001_21810000dup	Female	20.22	151.75	5.27	20 M	seq [GRCh37]dup(20)(p13p11.23) chr20:g. 1_20000000dup

Note

GA, gestational age; MW, maternal weight; NA, not available; M, million; FF, fetal fraction; Dup, duplication; ND, not detected; ATD(X), average target depth(X).

#### 3.2.3 Single‐gene disorders screening

**Figure 6:**
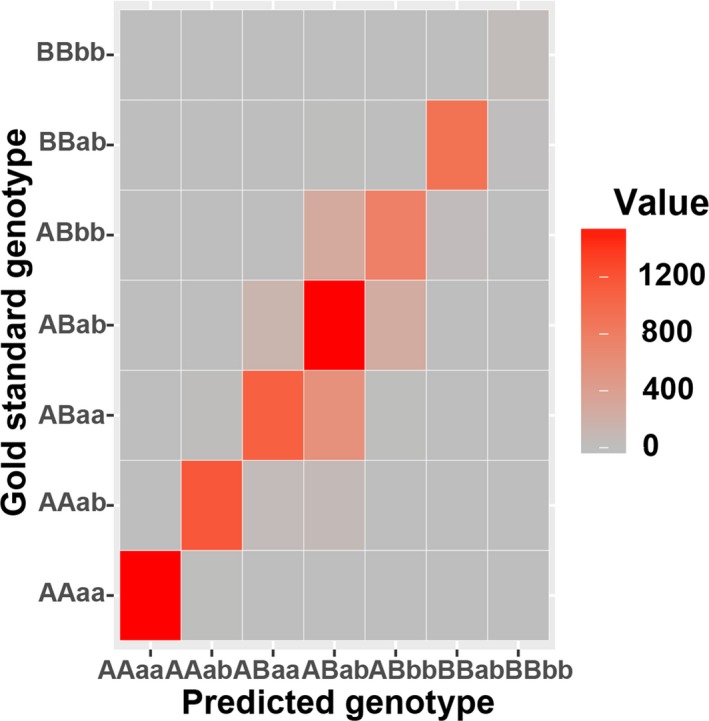
Comparison of predicted genotype and gold standard genotype. The combined genotypes derived from maternal plasma and fetal cord blood were used as gold standards. The accuracy of predicted genotypes was estimated with the standards. The value in the colorbar represents number of matched loci

To evaluate the performance of “pseudo‐tetraploid” model, the accuracy of genotype prediction was assessed by comparing seven estimated genotypes with the actual genotypes, which is obtained from the fetal cord blood and maternal plasma (Figure 6). Approximately 75% of loci were correctly determined and this demonstrated that the “pseudo‐tetraploid” method could be used to detect fetal SNPs. In this study, 12 out of 22 samples had a single‐gene disorder, in which, nine cases were correctly identified and three samples with one case of hearing impairment, one case of phenylketonuria, and one case of β‐thalassemia were missed by the test (Table 3). Therefore, the accuracy of fetus SNP detection was 86.4% (19/22) with 75% sensitivity (9/12).

**Table 3:** Comparison of single‐gene disorder identified using sanger sequencing of cord blood and targeted capture sequencing of maternal peripheral blood

Disease	Sample ID	Sanger sequencing of cord blood	NIPT targeted capture sequencing				
			Fetal gender	FF (%)	ATD(X)	PGC	Mutation
phenylketonuria	YFD01Y1401117‐1‐59	*PAH*:NM_000277:exon7:c.G728A:p.R243Q	Male	22.47	224.77	ABaa	ND
phenylketonuria	YFD01Y1401118‐1‐60	*PAH*:NM_000277:exon6:c.A611G:p.Y204C	Male	24.65	129.88	ABab	*PAH*:NM_000277:exon6:c.A611G:p.Y204C
hearing‐loss	YFD01Y1401108‐1‐50	*GJB2*:NM_004004:exon2:c.235delC:p.L79fs	Female	22.21	196.73	ABab	*GJB2*:NM_004004:exon2:c.235delC:p.L79fs
hearing‐loss	YFD01Y1401112‐1‐54	*GJB2*:NM_004004:exon2:c.G109A:p.V37I	Male	25.22	270.65	ABaa	ND
hearing‐loss	YFD01Y1401109‐1‐62	*GJB2*:NM_004004:exon2:c.299_300del:p.H100fs	Female	22.66	166.06	AAab	*GJB2*:NM_004004:exon2:c.299_300del:p.H100fs
		*GJB2*:NM_004004:exon2:c.235delC:p.L79fs				ABab	*GJB2*:NM_004004:exon2:c.235delC:p.L79fs
hearing‐loss	YFD01Y1401110‐1‐52	*GJB2*:NM_004004:exon2:c.299_300del:p.H100fs	Male	19.66	155.13	AAab	*GJB2*:NM_004004:exon2:c.299_300del:p.H100fs
		*GJB2*:NM_004004:exon2:c.235delC:p.L79fs				ABab	*GJB2*:NM_004004:exon2:c.235delC:p.L79fs
hearing‐loss	YFD01Y1401111‐1‐53	*SLC26A4*:NM_000441:exon10:c.A1174T:p.N392Y	Male	19.81	305.4	ABab	*SLC26A4*:NM_000441:exon10:c.A1174T:p.N392Y
β‐thalassemia	YFD01Y1401239‐1‐45	*HBB*:NM_000518:exon2:c.126_129del:p.F42fs	Male	21.18	130.05	ABaa	ND
β‐thalassemia	YFD01Y1401242‐1‐48	*HBB*:NM_000518:exon2:c.126_129del:p.F42fs	Male	21.35	201.09	ABab	*HBB*:NM_000518:exon2:c.126_129del:p.F42fs
		*HBB*:NM_000518:intron2:c.316‐197C>T				AAab	*HBB*:NM_000518:intron2:c.316‐197C>T
β‐thalassemia	YFD01Y1401224‐1‐61	*HBB*:NM_000518:exon2:c.126_129del:p.F42fs	Male	20.42	195.59	ABab	*HBB*:NM_000518:exon2:c.126_129del:p.F42fs
β‐thalassemia	YFD01Y1401227‐1‐70	*HBB*:NM_000518:intron2:c.316‐197C>T	Male	18.10	180.86	ABab	*HBB*:NM_000518:intron2:c.316‐197C>T
		*HBB*:NM_000518:exon2:c.126_129del:p.F42fs				AAab	*HBB*:NM_000518:exon2:c.126_129del:p.F42fs
β‐thalassemia	YFD01Y1401224‐1‐80	*HBB*:NM_000518:exon1:c.A52T:p.K18X	Male	21.03	241.74	ABab	*HBB*:NM_000518:exon1:c.A52T:p.K18X

Notes

FF, fetal fraction; ATD(X), average target depth(X); Del, deletion; ND, not detected; PGC, predicted genotypes combination.

OMIM accession number: Phenylalanine Hydroxylase (*PAH*), 612349; Gap Junction Protein, Beta‐2 (*GJB2*), 121011; Solute Carrier Family 26, Member 4 (*SLC26A4*), 605646; Hemoglobin‐‐Beta Locus (*HBB*), 141900.

## 4 DISCUSSION

The detection of cffDNA in maternal peripheral blood has been successfully employed in fetal trisomy screening, but aside from the common aneuploidies, other alterations such as sub‐chromosomal CNVs and specific single‐gene disorders are also prime concerns in clinical practice. So far, there has not been any reports on successfully detecting all three types of disorder within one NIPT test. Here, we carried out a proof of concept study to demonstrate the feasibility of integrating three tests into one single NIPT approach and our novel workflow could provide a foundation for a cheaper, faster, and multi‐functional noninvasive prenatal test in the future.

### 4.1 Panel design

The panel we designed in this study was based on the target region capture sequencing technology. By comparing with the whole genome sequencing, this approach could avoid genome regions of no functional significance and achieve a desired coverage of the targeted loci in a more cost‐effective way. Approximately 900 Kb of the reference genome was targeted in the panel and the inter‐probe distance was about 50 Kb. In this way, all the target regions could be ensured to have a high sequencing depth and the genotype of each hotspot locus could also be identified. Moreover, in consideration of the impact of GC bias, a consistent level of GC content was included in this panel to reduce variations in target hybridization and sequencing, and uniformly distributed regions with equal‐length and similar thermodynamic property would enable the comprehensive detection of chromosomal aneuploidy and sub‐chromosomal CNV.

### 4.2 Chromosome aneuploidy detection

NIPT autosomal aneuploidy detection has been widely used in routine clinical prenatal screening, and the sensitivities for trisomy 21, 18, and 13 have reached greater than 99% (Stokowski et al., 2015). However, for the sex chromosomes, the aneuploidy detection still remains problematic as indicated by several previous studies (Ramdaney, Hoskovec, Harkenrider, Soto, & Murphy, 2018; Reiss, Discenza, Foster, Dobson, & Wilkins‐Haug, 2017; Wang et al., 2014). Reiss et al. (2017) demonstrated that although NIPT had accurately predicted triple X and Klinefelter syndrome, it performed very poorly for fetal monosomy X detection. In our study, however, not only did we successfully identify the fetus with Klinefelter syndrome (47, XXY) but also a case of monosomy X (45, X) was picked out. Therefore, by combining with the autosomal aneuploidy detection, our approach seemed to have an improved overall accuracy rate and appeared to be able to detect monosomy X syndrome as well. However, larger sample set is still needed to further validate this observation.

### 4.3 Fetus CNVs screening

In general, factors that affecting the performance of CNVs detection include size, sequencing depth, fetal fraction, and GC content (Chen et al., 2013; Liu et al., 2016; Lo et al., 2016; Straver et al., 2014). In previous studies, the accuracy of detecting small fetus CNVs from maternal blood was always unsatisfactory. Li et al. (2016) showed that the detection rate dropped rapidly from 91% to 14% if the size of CNVs were below 5 M. Lo et al. (2016) evaluated the low‐coverage sequencing method for the detection of fetal CNVs and the total accuracy was only 64.5% (20/31) when 4–6 million reads were used to analyze CNVs with the size of 3 Mb to 42 Mb. Similarly, Yin et al. (2015) also showed that the performance was just 41.2% when CNVs were below 5 Mb. Unfortunately, in our study, despite the 100% detection rate of CNVs over 20 Mb, we too failed to identify CNVs smaller than 2 Mb. In addition, we discovered some size discrepancies between NIPT and amniotic fluid results (Figure 5, Table 2) and the difference was in mega bases (Mb). Since the judgment of pathogenicity primarily depends on the positions and sizes of CNVs, this size of inconsistency could potentially imply a significantly different clinical interpretation. Therefore, further improvements should be made on the aspects of target capture region quantity, probe density, locations of target region, and so on.

### 4.4 Fetus single‐gene disorder identification

Several studies have reported a relative haplotype dosage (RHDO) based method (Lo et al., 2010) to detect monogenetic diseases noninvasively through circulating single‐molecule amplification and re‐sequencing technology (cSMART). However, RHDO method needs to construct haplotype blocks surrounding unbalanced regions and cSMART tends to waste a large amount of data due to low efficiency in forming stable circular structure (Ge et al., 2013; Han et al., 2017; Lam et al., 2012; Lun et al., 2008; Lv et al., 2015), most importantly, RHDO approach could not be integrated into aneuploidy or CNV detection workflow. Therefore, we used a novel “pseudo‐tetraploid” model instead in our study to estimate the fetus genotype. We demonstrated that the overall consistency between predicted genotypes and the real ones was 75% and the test of 22 clinical samples showed an 86.4% overall accuracy (19/22). However, it is still difficult to distinguish the fetus ABAB (carrier) genotype from ABBB genotype (affected) in autosomal recessive diseases when mother is a heterozygous carrier because theoretically allele coverage of the heterozygous site should have a strong equilibrium of 1:1 ratio, but when the fetal fraction is low, the expected deviation will be below 0.5 and therefore it becomes difficult to separate true signals from random variations. This phenomenon of unexpected deviation from equilibrium is relatively universal in next‐generation sequencing (Newman et al., 2016; Zong, Lu, Chapman, & Xie, 2012). In addition, a report by Chen (Chen, Liu, Evans, & Ettwiller, 2017) indicated that mutagenic DNA damage could be a dominant factor for the erroneous identification of variants with low fetal frequency (1%–5%). Sequencing errors brought by the DNA damage existed in widely used databases, such as the 1,000 Genomes Project.

In conclusion, we have successfully integrated the detection of three different types of disorder into just one multi‐functional noninvasive prenatal test. The performance was strong in identification of chromosomal aneuploidy and CNVs over 20 Mb, but further optimizations are still needed for CNVs below 20 Mb and fetus SNP estimation. Possible directions include but not limit to, probe optimization to improve coverage uniformity, and denser probes surrounding genetic hotspots to enable haplotype analysis for increased genotyping accuracy, by incorporating linkage information of multiple loci. Furthermore, larger cohort of clinical samples should be used to validate this approach in the future.

## CONFLICT OF INTEREST

The authors declare no conflict of interest.

## AUTHOR CONTRIBUTIONS

Y.L. and B.J. designed research, performed experiment, analyzed data, and wrote the paper, K.Y., S.L., X.S., M.C., F.J., Y.D., J.W., Y.H., and S.C. performed some of the experiments. D.L and M.D. supervised this project, designed research, analyzed data, and wrote the paper.
